# Editorial: Plant Science's Contribution to Fighting Viral Pandemics: COVID-19 as a Case Study

**DOI:** 10.3389/fpls.2021.824440

**Published:** 2022-01-14

**Authors:** Linda Avesani, Fernando Ponz

**Affiliations:** ^1^Department of Biotechnology, University of Verona, Verona, Italy; ^2^Centro de Biotecnologia y Genomica de Plantas (CBGP), Madrid, Spain

**Keywords:** molecular farming, plant secondary metabolites, biofactories, botanic pharmaceutical, anti-virals

The use of plants to treat human diseases can be traced back to 6,000 years ago. The enormous biochemical arsenal of plants known as secondary (or specialized) metabolome has enabled the production of medicinal compounds to cure and/or ameliorate the symptoms of many human and animal diseases that are still prevalent today.

The enormous progress of plant biotechnology in the last 30 years has further boosted the potential of plants to help us cope with diseases, thanks to the advancement of molecular pharming, i.e., the use of plants to produce, by transient or stable transformation, biopharmaceuticals.

Given the current pandemic caused by severe acute respiratory syndrome coronavirus 2 (SARS-CoV-2), the international scientific community has gathered together in an urgent search for solutions to the current coronavirus disease-2019 (COVID-19) outbreak. To date, the plant-derived compounds chloroquine phosphate and artemisinin, which were previously used against malaria, have been repurposed for the treatment of COVID-19. Medicago is developing a candidate vaccine in plant factories, based on Virus-Like Particles (VLPs) of SARS-CoV-2, that is currently in phase III of its clinical trial, and promises to be one of the first subunit vaccines to enter the market. Furthermore, ongoing research supports the use of plants as bio-factories for the production of eagerly awaited SARS-CoV-2 monoclonal antibodies for passive immunization, which is meant to manage the disease.

In this emergency scenario, our topic aims to gather all relevant information on plant sciences to fight the COVID-19 pandemic by stimulating a “plant scientific hub” that builds on the enormous potential of plant science to contribute effectively to fighting present and future pandemics.

The current Research Topic includes six reviews, one mini review, one perspective, two opinion articles, and four original research studies focusing on (i) natural plant products, covering all the steps from production to extraction of bioactive molecules from medicinal plants, and on (ii) recombinant expression dissecting the use of plant molecular pharming for the production of viral recombinant proteins as diagnostic reagents, vaccines, and antibodies.

The former aspect is investigated in the four reviews, one mini-review, and two opinion articles.

In their review, Garcia provides a historical perspective on the use of plants in coping with pandemics, focusing on therapeutic use.

Another review by Pandey et al. focuses on the strategic use of phyto-inibitors to cope with the current pandemic in a way that could develop remedies for COVID-19 in a time-effective way.

In the mini-review of Matveeva et al., the authors summarize information on methods and approaches to searching for plant compounds including cheminformatics, bioinformatics, genetic engineering of viral targets, and others. As the authors discuss, this summary serves as an inspiration for experimental designs and to provide guidance for newcomers in the field.

In their review, Khan et al. review the potential of different *in vitro* plant cultures to produce medicinally important secondary metabolites to fight COVID-19.

Javed et al., Lucas et al., and Clark and Taylor-Robinson, respectively, in a review and in two opinion articles, focus on specific plant species and secondary metabolites, providing an overview of their potential for managing COVID-19 based on previous *in vitro* and *in vivo* studies. Specifically, carvacrol (Javed et al.), extracts of hops, Ceylon cinnamon (Lucas et al.), and pheophorbide *a* (Clark and Taylor-Robinson), a chlorophyll derivative, are considered in detail.

Plant molecular pharming is the main object of the remaining two reviews, perspective, and four original research articles.

In their review, Tusé et al. frame the use of plant molecular pharming from a perspective of feasibility by considering its potential from manufacturing to the current regulatory limitations and by comparing it to conventional manufacturing platforms based on mammalian cell cultures.

In this framework, Lico et al. in a perspective article, point out how molecular pharming could help in dealing with the current pandemic by focusing on Italy as a case study and the investment required in molecular pharming infrastructures. They define a roadmap for the development of diagnostic reagents and biopharmaceuticals to cope with COVID-19.

Poghossian et al., in their review, explore in detail the possibilities of plant virus-derived nanoparticles for the enhancement of virus biodetection based on field-effect devices (FEDs) used as biosensors. Previously, a brief perspective on recent advancements in the technology associated with FEDs has been provided.

In the four original research articles present in our Research Topic, some practical examples of the potential of plant molecular pharming to tackle SARS-Co V-2 are reported.

A complete and elegant description of the potential of plant molecular pharming is given by Diego-Martin et al., who describe production of milligram amounts of six different monoclonal antibodies and the receptor binding domain (RBD) of the Spike protein in *Nicotiana benthamiana* plants in the framework of an academic laboratory in just a few weeks, highlighting one major advantage of this approach, its speed.

Diagnostic reagents, meant to build up serological test for detecting the presence in sera of antibodies toward SARS-Co V-2, are the main focus of the research articles by Williams et al. and by Makatsa et al., respectively producing in plant systems the C-terminal half of SARS-CoV-2 N protein, and the S protein and RBD.

Plant-made therapeutic agents are described by Siriwattananon et al., that transiently produced Ace2 fused with the Fc region of human IgG1 assessing *in vitro* its potent neutralization capacity toward SARS-CoV-2, thus making the protein a therapeutic candidate for COVID-19.

## Author Contributions

Both authors listed have made a substantial, direct, and intellectual contribution to the work and approved it for publication.

## Conflict of Interest

The authors declare that the research was conducted in the absence of any commercial or financial relationships that could be construed as a potential conflict of interest.

## Publisher's Note

All claims expressed in this article are solely those of the authors and do not necessarily represent those of their affiliated organizations, or those of the publisher, the editors and the reviewers. Any product that may be evaluated in this article, or claim that may be made by its manufacturer, is not guaranteed or endorsed by the publisher.

